# 1918 Influenza, a Puzzle with Missing Pieces

**DOI:** 10.3201/eid1802.111409

**Published:** 2012-02

**Authors:** David M. Morens, Jeffery K. Taubenberger

**Affiliations:** National Institutes of Health, Bethesda, Maryland, USA

**Keywords:** influenza, influenza virus, viruses, pandemic, immunopathogeneis, bacterial pneumonia, epidemiology, 1918

Shanks and Brundage offer thought-provoking hypotheses about influenza pathogenesis during the catastrophic 1918–1919 pandemic (*1*). Although we neither agree nor disagree with their views, its central hypothesis of T-cell–mediated immunopathogenesis begs examination of past events in light of modern immunologic and virologic understanding. We also emphasize that effects of the pandemic virus should not be measured only by illness and death in 1918–1919, but should also take into account disease caused by its descendent seasonal and pandemic influenza viruses up to the present (*2*). Thus, for human influenza history to be better understood, it must be continually reevaluated.

Specifically, Shanks and Brundage hypothesize that high mortality rates in 1918 resulted from immunopathogenic effects of cell-mediated immune responses elicited by previously circulating influenza viruses. They also suggest that clues to immunopathogenic mechanisms are found in the unique, well-documented, W-shaped age-specific mortality curve of the 1918 pandemic (*3*) (Figure) in which the typical (U-shaped) curve of pandemic influenza, featuring mortality rate peaks in young and old persons, was augmented by an unprecedented third mortality rate peak in persons 20–40 years of age.

**Figure:**
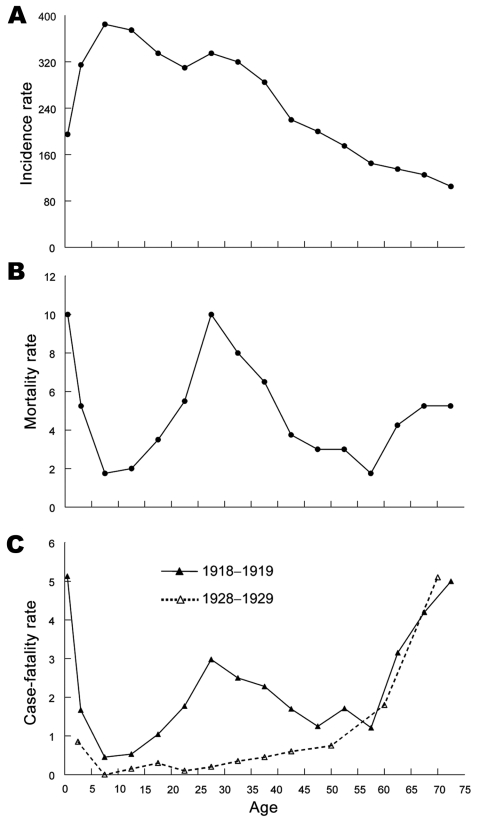
Combined influenza plus pneumonia (P&I) age-specific incidence, mortality, and case-fatality rates, per 1,000 persons/age group, US Public Health Service house-to-house surveys, 8 states, 1918, and US Public Health Service surveys during 1928–1929. A) P&I incidence for 1918; B) mortality rate for 1918 (ill and well persons combined); C) P&I case-fatality rates for 1918 (solid line) compared with a more typical curve of age-specific influenza case-fatality rates (dotted line) from 1928–1929. Reprinted from (*3*).

A complicating fact about 1918–1919 mortality patterns and pathogenesis hypotheses is that for ≈98% of infected persons, influenza was clinically unremarkable in its traditional signs and symptoms (fever, cough, myalgia) and severity (*4*). Clinical and epidemiologic differences were confined to 2 aspects: higher frequency of its long-appreciated post-illness complication—bacterial pneumonia (*5*)—and an unusual peak in fatal or nonfatal pneumonia cases in persons 20–40 years of age. In 1918, a higher percentage of persons of all ages, and especially those 20–40 years old, experienced influenza that led to cases of secondary bacterial pneumonia, which were caused by highly prevalent pneumopathogenic bacteria (especially pneumococci, streptococci, and staphylococci). These bacteria had been continuously causing primary pneumonia and pneumonia after influenza and other respiratory illnesses, and had long been exacting a substantial death toll.

These 1918 postinfluenza cases of pneumonia produced case-fatality rates similar to those of noninfluenza pneumonia caused by the same organisms. Moreover, antibacterial vaccines administered in 1918–1919 seem to have prevented postinfluenza deaths (*6*). Influenza mortality rates in 1918–1919 were most strongly associated with increased case incidence of, not increased severity of, common complicating bacterial pneumonia, and this finding was seen especially in persons 20–40 years of age. The epidemiology of 1918 influenza mortality is predominantly, almost entirely, the epidemiology of a single postonset complication: secondary bacterial pneumonia. Therefore, pathogenesis theories of severe or fatal 1918 influenza must account for why the 1918 virus predisposed more persons to secondary bacterial pneumonia, and also look beyond the virus to address bacterial cofactors. The hypotheses of Shanks and Brundage should be considered with these observations in mind.

An interesting aspect of the epidemiology of fatal 1918 influenza is demonstrated by epidemics in US military training camps, in which increased mortality rates were strongly associated with carriage epidemics of pneumopathogenic bacteria (*5*). An eerily analogous phenomenon had happened a year earlier (winter of 1917–1918) in deadly epidemics of measles/postmeasles bacterial pneumonia (*5*). Therefore, bacterial carrier status at the time of influenza virus introduction should be considered in interpreting mortality rate differences in soldiers and examined with respect to epidemiologic variables that could affect carriage (e.g., length of service, rural or urban differences, and health care worker status). Such simple exposure variables might explain at least some of the mortality rate differences pointed out by Shanks and Brundage.

With regard to possible immunoprotection afforded by earlier circulating influenza viruses, in our view, the picture is not fully interpretable. Epidemiologic information about the 1889 global pandemic suggests that the unidentified causative virus was novel in persons born after ≈1830 (*4*), if not before 1830. However, what the 1889 virus was, how long it may have circulated after 1889, in what form it may have drifted, and what level of population immunity in what age groups may have resulted are all speculative. Making various assumptions about post-1889 viral circulation patterns in an attempt to find epidemiologic evidence of protective or amplifying effects on incidence or mortality rates of 1918 influenza has not, to our knowledge, shown anything suggestive, let alone definitive.

Given that no age group in 1918 seems to have been protected by influenza exposures in 1889, some 1918 data are consistent with partial protection in persons >60 years of age (i.e., alive during and after the influenza pandemics of the 1830s and 1840s), even though the viruses involved in these pandemics had no discernible effect on 1889 influenza incidence (*4*). To further complicate the picture, major antigenic changes in the 1889 pandemic virus around 1900 have been postulated on the basis of epidemiologic/serologic evidence, and data from the 1957 (H2N2) and 1968 (H3N2) pandemics are each consistent with partial protection in persons alive during 1889–1918. Taken together, this information produces more questions than it answers, which suggests that only further virologic or serologic evidence based on examination of specimens from an earlier era can clarify the situation.

A related issue addressed by Shanks and Brundage concerns interpreting data on protection during the fatal October–November 1918 fall wave by influenza viruses circulating earlier in 1918 (we avoid the term spring wave on the grounds described below). In the 9 months before the 1918 fall wave, from which influenza (H1N1) viruses have been sequenced, 2 seemingly different types of influenza phenomena were observed. The first phenomenon was in January–May 1918 when scattered, explosive local outbreaks and epidemics of influenza-like illness occurred in various locations in Europe, and episodic outbreaks occurred in several other countries, which in virtually all cases showed lower than expected mortality rates for influenza. (Shanks and Brundage classify this spring activity, along with summer activity, as a spring wave.) If this wave was influenza, it was not a wave as the term had been used since 1889 to indicate global pandemic mortality.

The second phenomenon was a wave of moderate mortality rates that occurred not in the spring of 1918, but in the summer (July–August), mostly in a few countries in northern Europe. This summer wave seems consistent with a first major occurrence of the 1918 virus (H1N1), which may have found a tenuous foothold in the normally unfavorable summer months, predominantly in northern climes where temperature and humidity might be less restrictive of virus circulation. If this wave was the 1918 pandemic virus, simple arithmetic dictates that to have reached moderate explosiveness by July it must have been circulating for at least many weeks beforehand (*7*). Prepandemic circulation of virus (H1N1) in early 1918 could have caused at least some circumscribed outbreaks that elicited protection. However, if all winter–spring prepandemic 1918 activity had been caused by the pandemic virus, we are left with the conundrum of why it did not become pandemic then, when environmental circumstances were seemingly more favorable, and when it was being locally transmitted within the war zone in Europe at more explosive levels than the fall wave pandemic virus would later be. We must also explain the frustratingly contradictory protection data from spring or summer influenza-like illness during the fall occurrence of influenza.

Astute observers of the time considered the 1918 protection data uninterpretable (*8*). Because influenza viruses of different subtypes are now understood to protect against each other for prolonged periods (e.g., H1N1 against H2N2 and H2N2 against H3N2), interpreting 1918 protection data has become even more problematic. One or more viruses unrelated to the fall wave virus (H1N1) (e.g., an 1889 viral descendant) may have caused at least some of the observed protection and nonprotection phenomena in 1918. Less plausibly, the pandemic virus could have lost transmissibility while gaining pathogenicity after early 1918. However, in the absence of virologic evidence, the identity of early 1918 viruses that may have caused or failed to cause protection remains speculative.

Finally, despite whatever degree of immunopathogenesis or immunoprotection may have occurred in 1918, we see no particular reason to focus hypotheses on T-cell immunity over immunity conferred by antibody to viral antigens. The extremely high 1918 influenza infant mortality rate cannot easily be linked to cell-mediated immunity because infant T cells would presumably have never been exposed to influenza viruses. It is also noteworthy that mortality rates across the entire 1918 age spectrum were higher than in any other year between 1889 and the present time. In looking at the W-shaped mortality curve, we believe that the findings are striking for persons ≈5–14 years of age, the age range of persons with the lowest mortality rates in virtually all influenza pandemics and epidemics studied to date. In 1918, this age group appears to have had an ≈4-fold higher mortality rate than in 1889, conceivably indicating inherent viral virulence or, more correctly, viral–bacterial copathogenicity because most of the relatively few deaths in this age group seem also attributable to secondary bacterial pneumonia.

Although it is intriguing to speculate about the role of severe and fatal primary viral pneumonia, we are unaware of data suggesting that primary viral or viral immunopathogenic mechanisms accounted for high mortality rates in any 1918 age group; results of reported experimental animal studies are of uncertain relevance for humans. Almost all of the tens of thousands of autopsies reported in 1918 indicated classic bacterial pneumonia as the most prominent feature, which was different in frequency, but not in kind, from the familiar cases of pneumonia seen year in and year out, before and after 1918 (*5**,**7*). The data appear most consistent with some unidentified property of the 1918 virus (e.g., respiratory cell cytopathicity) that potentiated pneumonia with common bacteria carried in the upper respiratory tract (*5*). The cause of the middle peak of the W-shaped mortality curve remains a fascinating mystery that so far seems inexplicable by any hypothesis.

In summary, Shanks and Brundage have addressed 3 major mysteries of the 1918 influenza pandemic: high mortality rates/unexplained pathogenesis, unexplained age-specific mortality rate patterns, and evidence for wave-to-wave protection, with a unifying hypothesis. In our view, they justifiably point out that highly inconsistent wave-to-wave protection data from different 1918 observers represent essential clues to what happened 94 years ago. However, these clues have not yet led to satisfactory answers. They also draw attention to the W-shaped age-specific mortality curve, still unexplained we would argue, and hypothesize that it, as well as disease pathogenesis and protection, results from cell-mediated immune responses. Although we are not fully persuaded by all aspects of this hypothesis, it does suggest avenues for experimental and perhaps serologic and immunologic research. It should also stimulate us to rethink old mysteries in light of modern and evolving understanding of influenza. Questions about 1918 persist, and critical pieces of the puzzle, in our view, are still missing.
